# Alignment-free *k*-mer-guided design of a pan-*Orthoflavivirus* RT-qPCR assay

**DOI:** 10.1128/jcm.00388-26

**Published:** 2026-07-27

**Authors:** Khanate Sayasit, Chutikarn Chaimayo, Warinya Nuwong, Tharathip Boondouylan, Nattaya Tanlieng, Kamol Suwannakarn, Intawat Nookaew, Navin Horthongkham

**Affiliations:** 1 Department of Microbiology, Faculty of Medicine Siriraj Hospital, Mahidol University 65106, Bangkok, Thailand; 2 Department of Biomedical Informatics, University of Arkansas for Medical Sciences 12215, Little Rock, Arkansas, USA; Vanderbilt University Medical Center, Nashville, Tennessee, USA

**Keywords:** alignment-free, *k*-mer, RT-qPCR assay, primer-probe, *Orthoflavivirus*

## Abstract

The co-circulation and rapid expansion of the genus *Orthoflavivirus*, including dengue virus (DENV), Zika virus (ZIKV), West Nile virus (WNV), Yellow fever virus (YFV), and Japanese encephalitis virus (JEV), pose significant global health challenges. Developing inclusive pan-genus molecular diagnostics is hindered by high nucleotide divergence (>25–30%) and the computational limitations of traditional multiple sequence alignment in detecting conserved motifs across large data sets. To overcome these limitations, we developed a systematic alignment-free design pipeline that uses rigorous *k*-mer analysis and compacted De Bruijn graphs. We analyzed 11,846 RefSeq viral genomes to identify phylogenetically conserved, functionally relevant signatures within the *Orthoflavivirus* genus as a case study. The pipeline identified a conserved 600 bp region within the non-structural protein 5 gene, facilitating the design of a broad-spectrum TaqMan RT-qPCR assay. Analytical validation demonstrated limits of detection (LODs) of 1–10 copies/µL for DENV1–4, ZIKV, JEV, and YFV, with no cross-reactivity against non-target pathogens; WNV was consistently detected at 1,000 copies/µL in pilot experiments. In a clinical evaluation of archived samples, the assay achieved 97.33% overall accuracy. It demonstrated 100% sensitivity and specificity for DENV serotypes, yielding significantly earlier cycle threshold (Ct) values compared to a standard commercial kit, while ZIKV detection showed 100% specificity with 71.43% sensitivity. This study validates an alignment-free, *k*-mer-guided strategy for uncovering conserved diagnostic targets in highly variable viral genera, followed by local MSA-assisted primer-probe design. The resulting assay offers a robust tool for broad frontline surveillance, and the computational framework provides a scalable solution for future pandemic preparedness.

**IMPORTANCE:**

Mosquito-borne viruses like dengue, Zika, and West Nile pose a massive threat to global health. However, because these viruses are highly diverse and mutate rapidly, creating a single diagnostic test to detect all of them has been a challenge. Traditional software struggles to find shared genetic targets across such different viruses. To solve this, we developed a new computational approach. By analyzing over 11,000 viral genomes, we successfully identified a shared genetic signature of a group of mosquito-borne viruses. We used this discovery to create a single, highly accurate test capable of detecting multiple dangerous mosquito-borne viruses at once. This breakthrough provides a crucial, ready-to-use tool for frontline clinical diagnosis and global outbreak surveillance. More importantly, our strategy can be quickly adapted to design broad-spectrum tests for other rapidly mutating viruses, significantly strengthening our ability to prepare for and respond to future pandemics.

## INTRODUCTION

Arthropod-borne viruses (arboviruses) have been a major and expanding global health threat. Members of the genus *Orthoflavivirus*, including dengue virus (DENV), Zika virus (ZIKV), Japanese encephalitis virus (JEV), West Nile virus (WNV), and yellow fever virus (YFV), are responsible for recurrent epidemics, severe clinical manifestations, and substantial socioeconomic burden across tropical and subtropical regions (1). In 2024, more than 14 million dengue cases and over 10,000 dengue-related deaths were reported globally, underscoring the escalating worldwide burden of this arboviral disease (2). Asia, particularly Southeast Asia, bears a disproportionate share of the global dengue burden (3, 4). Co-circulation of multiple *Orthoflavivirus* species, rapid geographic expansion, and climate-driven changes in vector distribution have increased outbreak frequency and risk of large-scale epidemics. Climate warming is expanding the geographic range of *Aedes aegypti* (a primary vector of DENV and ZIKV, and the urban-cycle vector of YFV) into higher latitudes and altitudes, while *Culex* species (the mosquito vector for JEV and WNV) responses remain variable. Such expansion threatens to extend flaviviral transmission zones beyond historically endemic tropical areas into temperate regions. In October 2024, the World Health Organization launched the Global Strategic Preparedness, Readiness, and Response Plan for dengue and other *Aedes*-borne arboviruses, emphasizing diagnostic innovation as a critical priority for surveillance and outbreak response (5–8).

Timely and accurate detection is essential for effective surveillance and clinical management of co-endemic *Orthoflavivirus* infections; however, current diagnostic modalities have significant limitations. Serological assays are inexpensive and scalable but suffer from extensive cross-reactivity among flaviviruses, particularly IgG responses in populations with prior exposure or sequential infections, where secondary immune responses confound case attribution. Neutralization testing remains the gold standard for serological differentiation but is impractical for routine surveillance due to biosafety and technical constraints (9). While whole-genome next-generation sequencing provides unbiased detection and genomic information, it remains costly, slow, and technically demanding for routine frontline screening for clinical diagnosis in low- and middle-income settings. In practice, real-time reverse transcription quantitative PCR (RT-qPCR) has been the primary tool for acute-phase diagnosis and high-throughput surveillance. However, designing pan-*Orthoflavivirus* RT-qPCR assays that retain broad inclusivity across >25%–30% nucleotide-level divergence among distant species while avoiding off-target amplification represents a non-trivial challenge (10–12). Standard design approaches often fail to simultaneously achieve inclusivity across genetic diversity, specificity against non-target sequences, and robustness against emerging variants not represented in reference databases. Broadly inclusive, genus-level assays that capture phylogenetic diversity while enabling subsequent species- or serotype-level characterization are particularly attractive for surveillance networks in co-endemic regions (11). A pan-viral primer-probe set reduces the cost and complexity of initial screening compared to testing multiple species-specific RT-qPCR assays. While species-specific tests offer high analytical sensitivity and specificity, they increase reaction volume and multiplex complexity when screening for unknown causative agents. Therefore, samples testing positive via the pan-*Orthoflavivirus* assay should undergo subsequent characterization using species-specific RT-qPCR or sequencing for definitive identification.

Traditional assay development workflows rely on multiple sequence alignment (MSA) of reference genomes followed by heuristic identification of “conserved” regions for primer-probe placement. This approach carries intrinsic limitations. Even though modern MSA algorithms are computationally robust, the fundamental challenge is distinguishing genuine phylogenetically conserved features from alignment artifacts, especially in regions with competing sequence motifs or low information content. Moreover, generating and maintaining high-quality MSAs becomes increasingly challenging for ultra-large and/or taxonomically broad data sets as sequence databases expand. In practice, conserved-region identification is often biased toward well-studied reference strains (e.g., classical DENV1 Thailand strain and West Nile NY99), under-representing emerging lineages such as dengue genotype V (spreading across Brazil and South America as of 2024), recent Asian Zika genotype variants, or understudied tick-borne species (Powassan and Langat viruses) with limited representation. Together, these factors risk producing nominally “pan-genus” assays with hidden blind spots that show performance decay as novel variants accumulate (13–18). Alignment-free (AF) methods using *k*-mer analysis offer scalable alternatives for large-scale genomic data analysis (19, 20). However, researchers have employed AF approaches for whole gene and genome comparison by generating global similarity metrics or phylogenetic signals without consideration of local *k*-mer variation for designable primer-probe targets (21–24).

Thus, in this study, we developed a systematic pipeline for the identification of conserved regions, based on AF, *k*-mer-guided design for high-throughput multiplex RT-qPCR that targets the genus *Orthoflavivirus*. Our approach couples AF *k*-mer discovery with feature-aware genomic localization and standardized region selection based on (i) average common feature (ACF) metric, which is used to identify *k*-mer length, yielding specific for *Orthoflavivirus* genus (identifying *k* = 19 for 51 orthoflaviviral genomes); (ii) compacted de Bruijn graph analysis, which is used to identify conserved *k*-mer “seeds” that are anchored to functional genomic feature annotations, which ensured primer-binding sites reside within genuinely phylogenetically conserved, functionally relevant regions rather than low-information segments that appear conserved due to alignment artifacts; and (iii) local MSA is applied only on the identified conserved region (~600 bps), downstream a design aid, avoiding the brittleness of global MSA while still leveraging alignment information where it is reliable. We experimentally validated the designed primer-probe set using standard reference virus samples, and we applied the designed primer-probe set to clinical samples and compared the results with those of commercial kits.

## MATERIALS AND METHODS

### Data acquisition and preprocessing

The April 2025 FASTA nucleotide files for 18,678 representative viral genomes were downloaded from the NCBI RefSeq database via NCBI Virus, a portal for curated viral sequences and metadata (25). Taxonomic annotations according to the corresponding Taxonomic Identifier (TaxID) were generated with Taxonkit version 0.20.0 (26) using the NCBI Taxonomy dump (September 2025). Segmentation status (segmented vs non-segmented) was annotated using the ViralHostDB database (27). General feature format 3 (GFF3) annotation files were downloaded for further annotation during block calling.

To reflect biological reality, each genome was represented by a single FASTA file in <taxid>.fasta convention. The downloaded genome records were first filtered by whether they were non-segmented or segmented viruses. Non-segmented genomes were filtered by the keywords in the genome description, and only those with the keywords “complete genome,” “complete sequence,” and “genome” were included. If genomes shared the same taxid, they were assigned an accession number on the filename in the format <taxid_accession>.fasta format; otherwise, the filename was labeled as <taxid>.fasta. For segmented viruses, genomes with “partial” in the description, as well as viruses with only one record per TaxID, were discarded. Additionally, genomes that survived the keyword filtering step were kept, resulting in multi-FASTA files used in downstream analyses.

### Alignment-free phylogeny for clade visualization (Mash-style transformed Jaccard index)

To provide a fast overview of clade structure across *Flaviviridae*, we computed a pairwise distance matrix from preprocessed FASTA genomes using Mash distance, which was transformed from Jaccard index to estimate the mutation rate per site between two sequences as implemented in the *k*-mer-length iterative selection for unbiased ecophylogenomics (KITSUNE) dmatrix module. Briefly, genomes were converted to *k*-mer sets, the exact Jaccard similarity *J* was computed between *k*-mer presence/absence (PA) profiles, and distances were obtained via the Mash transform *D* = (−1/*k*)(ln(2*J*/1 + *J*)). The resulting distance matrix was used to infer a neighbor-joining tree, which can be used to cluster and visualize clades, not for deep phylogenetic inference (21). We used a fixed value of 11 for *k* to avoid parameter overfitting and to remain consistent with KITSUNE’s viral benchmarking, which reported *k* = 11 as an empirically optimal and subsampling-robust choice for viral comparisons. Conceptually, this value balances two competing failure modes: (i) when *k* is too small, unrelated genomes can share many short *k*-mers by chance, compressing distances and obscuring clade separation; (ii) when *k* is too large, overlap between divergent genomes becomes sparse, causing distances to saturate and become unstable. Using *k* = 11 maintains informative overlap for inter-genus comparisons while preserving separation of major clades (28). The tree was rooted using *Pestivirus* as an out-group to visualize in-group/out-group separation. Tips and clades were colored by genus (*Orthoflavivirus*, *Hepacivirus*, *Pegivirus*, *Pestivirus*, and unclassified *Flaviviridae*) to facilitate qualitative assessment of expected genus-level grouping. Bootstrap support values were not reported because this neighbor-joining tree is used for descriptive visualization rather than inferential phylogenetic conclusions; downstream analyses focus on conserved-window discovery and primer ability rather than resolving deep branching order (29).

### ACF-guided *k* selection and shortest unique *k*-mer plots (KITSUNE)

We used KITSUNE to guide *k* selection with the ACF, defined as the mean pairwise intersection size of canonical *k*-mer sets at length *k* (reverse-complement canonicalized). KITSUNE ACF can be used to find the maximum informative feature length of *k*-mers for AF feature frequency profile phylogenomic analysis (29), and can also identify the shortest unique *k*-mer (SUK) length for diagnostics with the following method: for a given query genome, sweep *k* and count how many reference genomes (excluding the query itself) share ≥1 exact *k*-mer with the query; the SUK is the smallest *k* for which this count is 0, indicating the presence of a species-specific signature in the database (28). Moreover, we can infer the length at which a degree of shared *k*-mers is present.

We performed *k*-mer counting with Jellyfish version 2.3.1, a fast and memory-efficient tool to count *k*-mers (30). In KITSUNE, the “--fast” option directs Jellyfish to count all *k*-mers (no frequency filter), whereas the default run applies Jellyfish’s high-frequency *k*-mer filter (a two-pass procedure).

We used KITSUNE ACF with the --fast option, so that we included all *k*-mers present in a genome, to query each *Flaviviridae* genome against the cleaned RefSeq reference set. We then subset the results to the genus *Orthoflavivirus* and plotted ACF vs *k* to assess within-group commonness. Counts were reverse-complement canonicalized, and *k*-mers overlapping non-ACGT characters were ignored.

### Analysis of *k*-mer intersection

For each *k*, KITSUNE ACF recorded every query-reference pair, whether ≥1 exact canonical *k*-mer was shared. We aggregated these per-pair records into per-*k* summaries. To reduce storage and complexity, we did not retain the identities of the intersecting *k*-mers at the global reference level. This is sufficient for all analyses that rely on PA. For downstream conserved-block discovery at *k*, we performed a separate, targeted re-count within the *Flaviviridae/Orthoflavivirus* set that does capture k-mer identities and supports block merging. If weighted overlaps were required, they were reconstructed from the released FASTA snapshots.

### Presence-absence matrix of *k*-mers

Using the *k*-mer length of 19 from the ACF step, genomes were decomposed into canonical 19-mers. We first precomputed Jellyfish *k*-mer count databases for the full viral collection and *the Orthoflavivirus* subset using Jellyfish count. We then used Jellyfish query/dump to compute exact *k*-mer membership against the prebuilt universe databases. This method yields a binary PA matrix (columns = 51 *Orthoflavivirus* genomes; rows = distinct universe-referenced 19-mers observed in the *Orthoflavivirus* set). From the PA matrix M, we quantified within-genus conservation for each *k*-mer as a *present_in* number (the number of *Orthoflavivirus* genomes containing the *k*-mer) and its fraction = present_in/*N*. We retained the list of genomes in which each *k*-mer occurred for downstream QC.

### Compacted De Bruijn graph construction

We used De Bruijn graphs to encode exact (*k* − 1)-base overlaps between *k*-mers, where each edge is a *k*-mer and connects two nodes that are its (*k* − 1)-prefix and (*k* − 1)-suffix. Long, perfectly consistent stretches of sequence appear in the graph as non-branching paths. By merging each maximal non-branching path into a single node (a unitig), the graph is compacted without loss of sequence information (31). To obtain compacted De Bruijn graphs and unitigs, we used Bifrost version 1.3.5, a highly parallel construction and indexing of colored and compacted De Bruijn graphs that implements a highly parallel algorithm for direct construction and indexing of colored compacted De Bruijn graphs (32). The unitigs that were derived from *k*-mers shared by at least three genomes (high-confidence unitigs) were retained for further steps.

### Exact mapping and feature annotation

We mapped the high-confidence unitigs to genomic coordinates by aligning them to a prebuilt Bowtie index of the orthoflaviviral genomes in exhaustive, exact-match mode, reporting all zero-mismatch hits as sequence alignment/map (SAM) records. Bowtie version 1.0.0 was chosen for its speed and strict mismatch control for short sequences (33). SAM alignments were converted to coordinate-sorted, compressed binary versions of SAM files (BAM files) with SAMtools version 1.16.1 (view, sort) and then to browser extensible data (BED) intervals (0-based, half-open) with BEDtools version 2.30.0 (34, 35), yielding genomic coordinates for each unitig placement. To link these coordinates to a functional context, we intersected the unitig BED intervals with per-genome GFF3 annotation using BEDtools intersect in strand-agnostic mode, annotating each hit with any overlapping feature.

### Conserved-window selection and scoring

We used a window length of 300 bp, treating this as a design window rather than the final amplicon. This length is approximately two to four times a typical optimal TaqMan RT-qPCR amplicon (∼70–150 bp), which is generally considered the maximum limit to ensure high efficiency and accurate quantification. It also provides enough local context to place primers and a probe, to tolerate modest sequence variation and small indels, and to accommodate differences in genome length, while still offering fine-scale resolution along the approximate 10–11 kb *Orthoflavivirus* genomes. Features shorter than 300 bp were taken in full as their conserved windows. For longer features, we generated candidate window starts at all unique unitigs hit boundaries within the feature (distinct hit starts, hit ends, and the feature start). For each candidate start, a 300 bp window was placed and, if necessary, shifted so that it remained entirely within the feature bounds.

For every window, we computed two scores: (i) the summation number of bp of the unitigs mapped on the individual 300 bp windows of the orthoflaviviral genomes, and (ii) the number of orthoflaviviral genomes that the unitigs mapped on the individual 300 bp windows. Candidate windows were therefore ranked primarily by the score for further selection of conserved region(s) that were used in the next step.

### Sequence extraction and multiple sequence alignment

For selected high-scoring regions, we extracted the corresponding nucleotide sequences using a 600 bp design region spanning the peak interval and its immediate flanks. This extraction length provides sufficient local context for primer-probe placement while accommodating genome-length variation and small boundary shifts across genomes. Extracted regions were written in a single multi-FASTA file containing sequences from all viruses, and multiple sequences were aligned with MAFFT version 7.505 using the L-INS-i algorithm, a high-accuracy method using local pairwise alignment (36).

### Consensus-guided primer design, degeneracy evaluation, and specificity assessment

MSAs of candidate conserved windows were manually inspected for the location of conserved identity blocks in Geneious Prime version 2026.0.1 to guide primer-probe placement. Because sequence diversity necessitated the use of degenerate oligonucleotides, IUPAC ambiguity codes were introduced conservatively, and total degeneracy was capped at ≤100 variants per oligo to maintain effective working concentration. A >50% per-position base frequency threshold was used to define the majority consensus base and guide local MSA inspection. Positions without a majority base were encoded with appropriate IUPAC ambiguity codes only when this did not exceed the degeneracy cap; otherwise, they were avoided during primer-probe placement. Thus, the >50% threshold guided consensus interpretation, whereas final oligonucleotide selection was constrained by local conservation, total degeneracy, 3′-end mismatch avoidance, primer/probe design criteria, secondary-structure filtering, and *in silico* specificity assessment.

Primer-probe design rules were applied to optimize amplification and detection efficiency. Forward primer placement was prioritized because mismatches near the 3′ end (defined here as the last five bases) can substantially reduce RT-qPCR efficiency; therefore, we preferentially selected regions with contiguous conserved bases and minimized degeneracy toward the 3′ end. In addition, forward primers were constrained to avoid >4 consecutive G residues and to limit G/C content near the 3′ terminus. Probe placement required a conservation level comparable to the forward primer, as mismatches near the probe center can markedly reduce hybridization and cleavage efficiency (37). Additional probe constraints included avoiding a 5′ G residue, avoiding >4 consecutive G residues, avoiding ≥6 consecutive A residues, and avoiding G at the second position from the 5′ end.

Because reverse transcription is primed by the reverse primer in this one-step assay, limited mismatches may be more tolerated during the RT step with a Moloney murine leukemia virus-derived reverse transcriptase; however, mismatches at the extreme 3′ end were still minimized because both primers must support efficient PCR amplification during cycling (38). Probe melting temperature (*T*_m_) was designed to be higher than primer *T*_m_, and primer *T*_m_ values were matched as closely as possible. Secondary structures were assessed using predicted hairpin melting temperatures and Δ*G* values; candidates with hairpins or dimers predicted to be stable, in other words, a strongly negative Δ*G*, and those with a structure *T*_m_ approaching the annealing temperature (52/55°C), were discarded. All degenerate primers and probes were expanded into explicit sequence variants and evaluated in IDT OligoAnalyzer in batch mode using reaction-relevant oligo and salt parameters (39). We then assessed the specificity of the designed primers and probe using BLASTN against the NCBI non-redundant nucleotide database, mimicking *in silico* PCR, and allowing ≤2 mismatches based on primers and probe sequences to approximate assay behavior. Because BLASTN does not model degenerate oligonucleotides as mixtures of all possible sequence variants, all degenerate primers and probes were expanded into explicit sequence variants before the search.

### Standard virus reference strains and RNA extraction

Reference strains of medically important arboviruses were used to evaluate the analytical specificity of the assay. The standard panel included DENV serotypes 1–4 (DENV1–4), ZIKV, chikungunya virus (CHIKV), JEV, WNV, and YFV. Reference viruses were obtained from the American Type Culture Collection (ATCC), including DENV1 strain Hawaii (ATCC VR-1856), DENV2 strain TH-36 (ATCC VR-1810), DENV3 strain H87 (ATCC VR-3380), DENV4 H241 (ATCC VR-1490), and ZIKV strain PRVABC59 (ATCC VR-1843). JEV strain P3 was obtained from the Faculty of Tropical Medicine, Mahidol University, Thailand. Nucleic acid controls for WNV strain New York-99 (MBC069-R) and YFV strain 17D (MBC100-R) were obtained from VIRCELL Molecular. All viruses except WNV and YFV were cultured and subjected to lysis before RNA extraction. RNA was extracted from 200 µL of supernatant using the MagLEAD 12gC automated extraction platform (Precision System Science) following the manufacturer’s protocol and eluted in 100 µL of RNase-free water. Extracted RNA was either used immediately or stored at −80°C for short-term use.

### Evaluation of analytical specificity using an arboviral standard viral panel and cross-reactivity testing

To assess the potential detection of the designed primers and probe, we tested RNA extracted from each reference virus using the developed real-time RT-qPCR assay. Real-time RT-qPCR was performed in a total volume of 20 µL using qScript XLT One-Step RT-qPCR ToughMix, Low ROX master mix (Quantabio). Each reaction contained 2× master mix, forward primer (500 nM), reverse primer (500 nM), probe (125 nM), and 5 µL of template. Nuclease-free water was added to reach the final volume. Reactions were run on a QuantStudio 7 Pro Real-Time PCR System (Thermo Fisher Scientific) under the following cycling conditions: cDNA synthesis at 50°C for 10 min, initial activation at 95°C for 1 min, followed by 45 cycles of denaturation at 95°C for 10 s and combined annealing/extension at 52/55°C for 60 s with fluorescence acquisition at the end of each cycle. Baseline and threshold values were automatically calculated by the instrument software and manually adjusted if necessary. Cross-reactivity testing was performed to assess the analytical specificity of the designed primer-probe set. The assay was evaluated in a single-reaction format using nucleic acids from the following non-target pathogens: influenza A virus H1N1 strain A/PR/8/34 (ATCC VR-95), respiratory syncytial virus (RSV) strain A2 (ATCC VR-1540), RSV strain B (ATCC VR-1580), CHIKV strain ROSS (Department of Medical Sciences, Ministry of Public Health, Thailand), enterovirus A71 (EV-A71) strain H (ATCC VR-1432), cytomegalovirus (CMV) strain AD-169 (ATCC: VR-538), hepatitis C virus (HCV), hepatitis B virus (HBV), human immunodeficiency virus (HIV) strain AE, *Orientia tsutsugamushi* strain KARP (BioSample accession SAMN51290297), and *Rickettsia typhi* strain SI-typh0423 (BioSample accession SAMN51290298). *Plasmodium falciparum* strain 3D7 (ATCC; PRA-405D), *Leptospira interrogans* serovar Copenhageni strain Fiocruz L1-130 (ATCC; BAA-1198D-5), *Escherichia coli* strain Seattle 1946 (DSM 1103, NCIB 12210); ATCC: 25922, *Staphylococcus* aureus strain Seattle 1945; ATCC: 25923, and *Pseudomonas aeruginosa* (ATCC: 27853). Each pathogen was tested ranging from 100 to 10,000 copies/µL, except HBV and HCV were tested at 2,550–2,600 IU/mL and 18,000–49,300 IU/mL, respectively.

### Determination of copy number

A kit-provided positive-control template (2 × 10^5^ copies/µL) (Primerdesign genesig advanced kits) was serially diluted 10-fold in Template Preparation Buffer (Primerdesign Ltd) to generate standards of 2 × 10^4^, 2 × 10^3^, 2 × 10^2^, 2 × 10^1^, and 2 copies/µL. For standard-curve generation, 5 µL of each dilution was added to 15 µL reaction master mix (final volume, 20 µL) containing the corresponding primer-probe set, corresponding to 10–10^5^ copies per reaction across the dilution series. Standard curves were constructed by linear regression of Ct values against log_10_ (copies per reaction). Standard-curve reactions for DENV, ZIKV, and JEV were performed using the SuperScript III One-Step RT-PCR System with Platinum Taq DNA Polymerase (Invitrogen) (see Fig. S1). The resulting standard curves were used to estimate the RNA copy number of extracted nucleic acids from the arbovirus reference strains based on their Ct values.

### Limit of detection, repeatability, and reproducibility of the designed primers and probe set

The limit of detection (LOD) and repeatability of the designed primer-probe set were evaluated using extracted total nucleic acids from eight arbovirus reference strains, including DENV1–4, ZIKV, JEV, YFV, and WNV. The LOD was defined as the lowest concentration yielding positive amplification in >95% of replicates under the experimental conditions. Any amplification signals observed at lower concentrations but not meeting the threshold were not regarded as the LOD. The real-time RT-qPCR reaction was performed as previously described. The standard deviation (SD) and coefficient of variation (CV) were calculated.

For DENV1–4, ZIKV, and JEV, the LOD and intra-assay repeatability were assessed in 12 replicates of various concentrations of copy numbers per microliter PCR reaction (1–10,000 copies/μL). Inter-assay reproducibility was evaluated by two technicians over 5 days, with a total of 20 replicates. Due to the limited nucleic acids of YFV and WNV, the LOD evaluation included initial screening experiments performed in triplicate (100–10,000 copies/μL). Additional lower-concentration testing was subsequently performed using 12 replicates at 1 and 10 copies/µL, if applicable.

### Pathogen-specific commercial kits used

Commercial assays were performed using the Primerdesign genesig advanced kits for ZIKV and DENV (Primerdesign Ltd). We performed the assays of the commercial kits, following the manufacturer’s protocols.

### Performance on clinical samples

We validated the sensitivity and specificity of the designed primer-probe set using 150 archived clinical samples with prediagnosed arboviral infection, including 25 plasma samples positive for DENV1, DENV2, and DENV4, 11 positive for DENV3, 14 positive for ZIKV, as well as 50 negative plasma samples. Prior to the designed primer-probe validation, all positive samples were confirmed using the pathogen-specific commercial real-time PCR assay (Primerdesign Ltd. genesig advanced kit), which was used as the standard test. All 50 negative samples were also confirmed to be negative for all flaviviruses. For the real-time analysis, 5 µL of extracted nucleic acids was mixed with 15 µL of mastermix (total volume of 20 µL) using qScript XLT One-Step RT-qPCR ToughMix (Low ROX) (Quantabio). Each reaction contained 2× master mix, forward primer (500 nM), reverse primer (500 nM), and 125 nM of probe. The real-time RT-qPCR was performed as previously described. The details of the archived clinical samples are provided in Table S1A and B.

### Statistical analysis

Ct values obtained with the designed primer-probe set were compared with those from the commercial kit. To summarize assay repeatability and the distribution of Ct values, we calculated the SD, as well as the median and interquartile range. We also reported the percentage coefficient of variation (%CV), which summarizes variability in Ct values across replicates. Diagnostic performance was assessed by calculating sensitivity, specificity, positive predictive value, negative predictive value, and overall accuracy, together with 95% confidence intervals (95% CIs), using MedCalc’s diagnostic test calculator (https://www.medcalc.org/calc/diagnostic_test.php). Bland–Altman plots were used to evaluate agreement between the two assays across the Ct range. Differences in Ct values between the designed primer-probe set and the commercial kit were assessed using a two-sided paired *t*-test, and Pearson correlation was used to assess the strength of the linear relationship between Ct measurements. Plots were generated in GraphPad Prism 10.

## RESULTS

**Fig 1:**
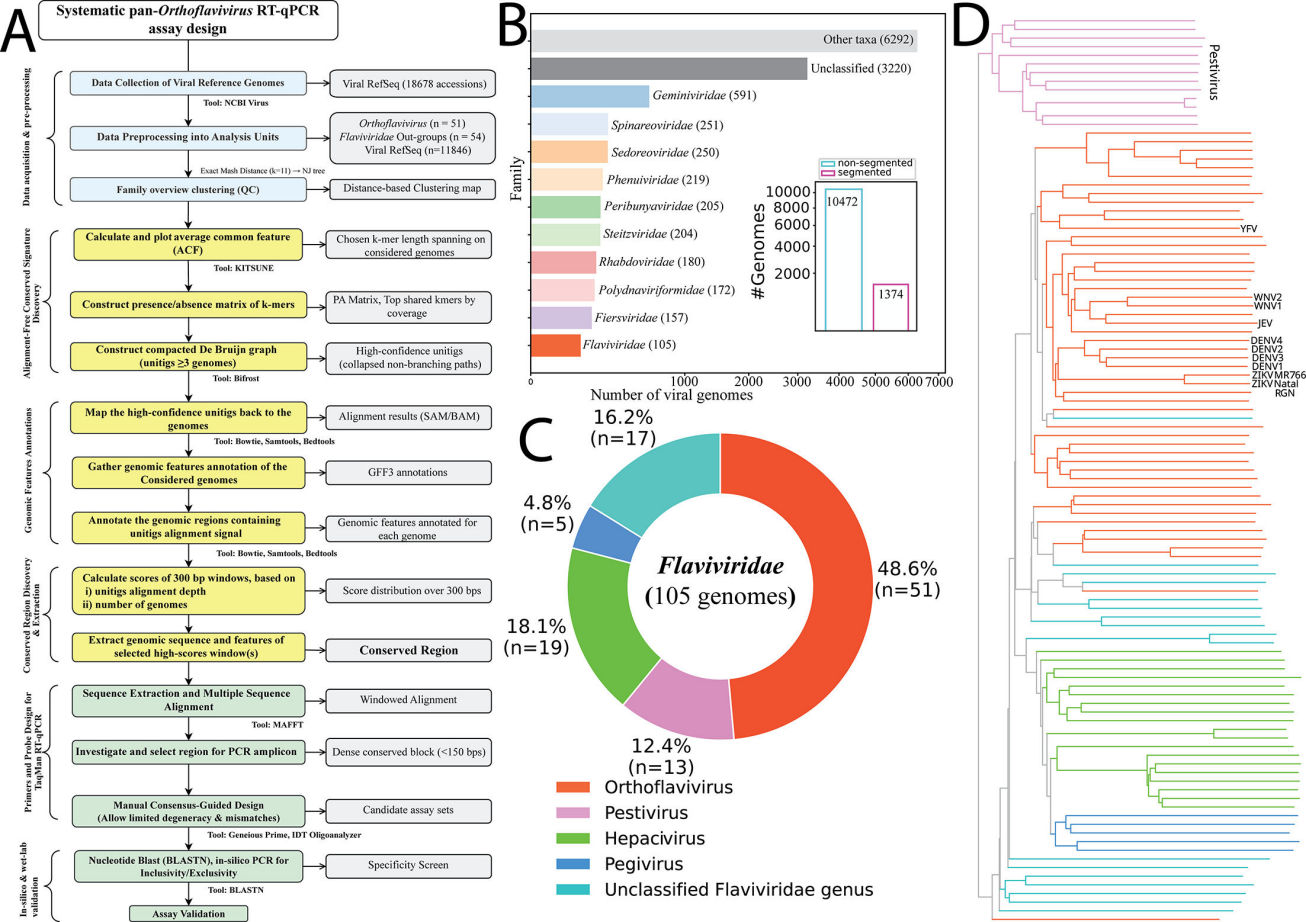
A comprehensive workflow for a pan-*Orthoflavivirus* RT-qPCR assay design, from data set curation and alignment-free conserved-window discovery for assay validation. (**A**) The workflow includes: (i) data set verification and quality control; (ii) alignment-free *k*-mer-guided discovery, localization, and ranking of feature-anchored windows; and (iii) local MSA-assisted manual primer-probe design followed by specificity screening. Box colors indicate workflow phases; gray boxes denote intermediate artifacts/outputs; yellow boxes mark workflow components introduced in this study. (**B**) Family-level representation of data set composition. Bars show the top 10 viral families by number of genomes, with *Flaviviridae* shown additionally for reference (highlighted), even though it falls outside the top 10. The cleaned data set comprises 11,846 viral genomes, including 10,472 non-segmented and 1,374 segmented genomes. Most genomes fall into other taxa (6,292) and Unclassified (3,220), followed by *Geminiviridae* (591), *Spinareoviridae* (251), *Sedoreoviridae* (250), *Phenuiviridae* (219), *Peribunyaviridae* (205), *Steitzviridae* (204), *Rhabdoviridae* (180), *Polydnaviriformidae* (172), *Fiersviridae* (157), and *Flaviviridae* (105). (**C**) Genus-level composition of the family *Flaviviridae* in the RefSeq complete/near-complete genome data set. *Orthoflavivirus* comprises the largest fraction (48.6%, *n* = 51), followed by *Hepacivirus* (8.1%, *n* = 19), unclassified *Flaviviridae* (16.2%, *n* = 17), *Pestivirus* (12.4%, *n* = 13), and *Pegivirus* (4.8%, *n* = 5). Genera are ranked by the number of complete/near-complete RefSeq genomes. (**D**) An alignment-free distance matrix was computed from preprocessed *Orthoflavivirus* genomes using a Mash-style transformed Jaccard metric (*k* = 11) and used to infer a neighbor-joining tree (rooted with *Pestiviruses*, magenta branch on the top). The topology recapitulates expected intra-genus structure and coherent species-level clades, which we used to verify taxonomic assignments and flag outliers prior to conserved-signature analysis. The focused species within this study were labeled with their names on the tree. DENV1–4 = dengue virus serotypes 1–4, JEV = Japanese encephalitis virus, WNV1,2 = West Nile virus lineage 1,2, ZIKV_MR766 = Zika virus, and ZIKV_ Natal_RGN = Zika virus. See Fig. S2 for complete genus phylogenomic context of *Orthoflavivir*us.

The workflow of our systematic primer and probe design for pan detection of *Orthoflavivirus* genus is summarized in Fig. 1A. The key steps of the workflow are described in the following sections.

### Data acquisition and preprocessing: viral genome data set composition and phylogenetic validation

We began by downloading 18,678 accession numbers of viral genomes from NCBI RefSeq with their taxonomic rank information in April 2025. After filtering out non-genome accession numbers, we were left with 15,467 accessions, comprising 10,472 non-segmented viral genomes and 1,374 segmented viral genomes. In total, 11,846 viral genomes were used for further analysis as a reference genome set. The distribution of the top 10 viral families by number of genomes is illustrated in Fig. 1B (all genomes and annotations are summarized in Table S2A and B). Focusing on the *Flaviviridae* viral family (Fig. 1C), 51 of 105 genomes were assigned to the *Orthoflavivirus* genus*,* and another 54 genomes belonged to other genera—*Pestivirus, Hepacivirus, Pegivirus,* and Unclassified—providing both in-group and closely related out-group context for conserved-signature discovery analysis. The AF phylogenomic relationships were reconstructed and illustrated in Fig. 1D (see Fig. S2 for complete genus phylogenomic context of *Orthoflavivir*us). The expected clustering of *Orthoflavivir*us genomes was clearly observed in the phylogenomic tree, supporting the taxonomic coherence of the data set used for downstream analysis. One exception was the Tamana bat virus (TABV), which was included based on the taxonomic annotation available at the time of data set construction. TABV formed a highly divergent branch relative to the main *Orthoflavivirus* cluster. This observation aligns with a previous comparative genomics study showing that TABV is closely related to flaviviruses (40). In addition, selected clinically important *Orthoflavivirus* species such as DENV1–4, JEV, WNV lineages 1 and 2, YFV, and two ZIKVs, marked on the tip of the phylogenomic tree, were used for experimental validations in the study.

### Alignment-free conserved sequence signature discovery: identification and graph-based consolidation of conserved *k*-mer signatures

**Fig 2:**
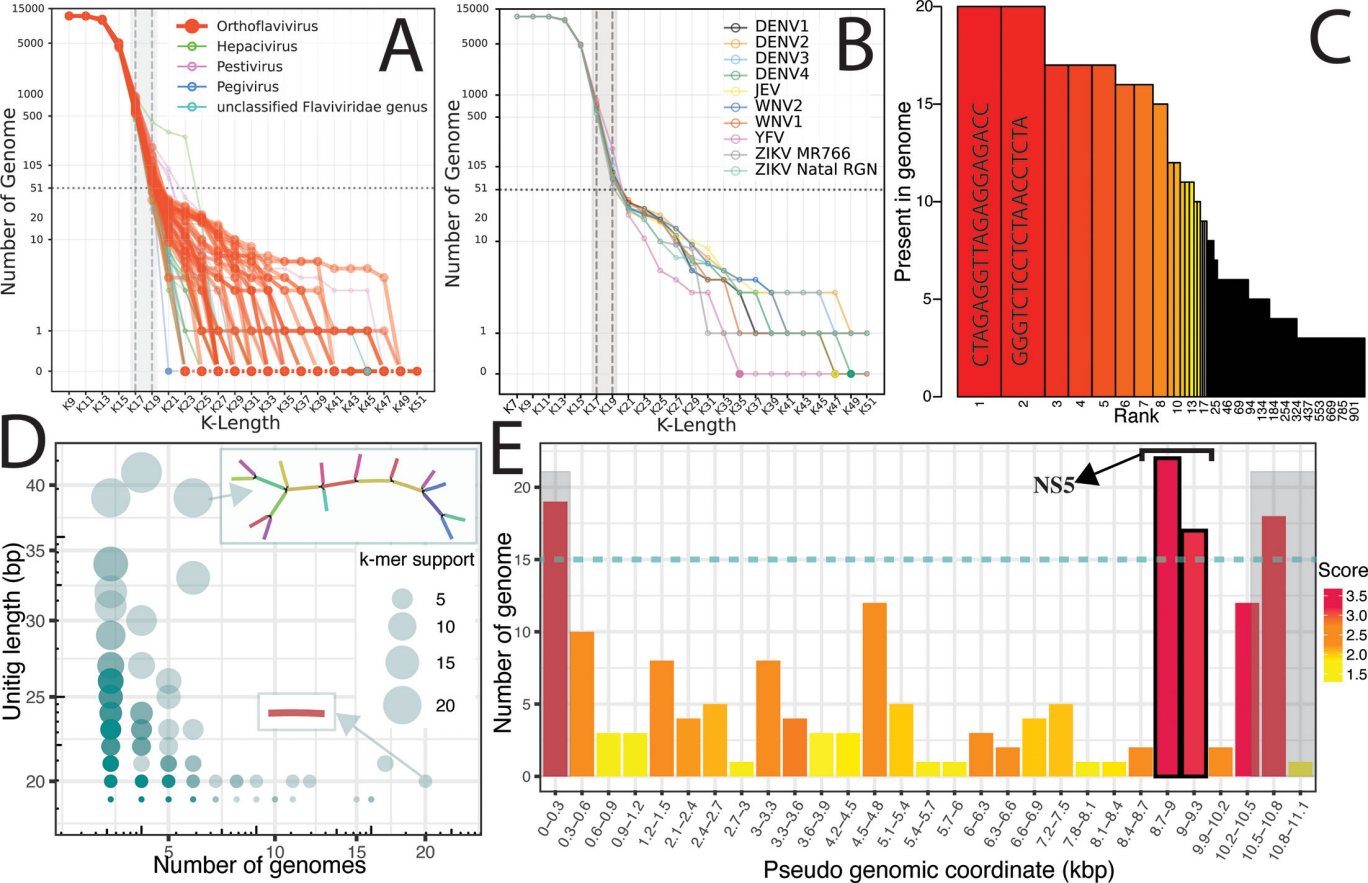
Alignment-free conserved-signature discovery and localization in *Orthoflavivirus* genomes. (**A**) Average common feature shortest-unique *k*-mer (SUK) curves across *k* = 9–51. Vertical guides highlight the onset of rapid feature loss from K17 and a sharper decline around K19, marking the transition from broadly shared to increasingly specific *k*-mer features. (**B**) SUK plot for the *Orthoflavivirus* considered genomes—dengue virus (DENV1–4), Japanese encephalitis virus (JEV), West Nile virus (WNV), yellow fever virus (YFV), and Zika virus (ZIKV)—showing the decay in shared *k*-mer features as *k* increases. We selected *k* = 19 for downstream conserved-signature discovery because it retained sufficient genus-level shared features, whereas at *k* ≥ 21 the number of shared *k*-mers dropped below the panel size (*n* = 51), indicating that longer *k*-mers became too specific for detecting features conserved across the full panel. (**C**) Genome-presence rank plot of 19-mers within *Orthoflavivirus*. Bars show the number of genomes containing each 19-mer sequence, ranked from highest to lowest; the top two highest-presence 19-mers are labeled. (**D**) Unitig summary after compacted de Bruijn graph (cDBG) consolidation of 19-mers filtered to those present in ≥3 genomes. Unitig length is plotted against the number of genomes supporting each unitig. Point size indicates *k*-mer support (number of distinct 19-mers per unitig). Highly conserved unitigs (supported by more genomes) are generally shorter, whereas longer unitigs are typically supported by fewer genomes. (**E**) Genome-wide conservation hotspot profile along a pseudo-genomic coordinate. Bars indicate the number of genomes contributing conserved hits per window, with color encoding conservation score. Prominent peaks coincide with the NS5 region; terminal repeat/UTR regions are indicated in gray (0–0.3 and 10.5–11.1 kbp). The gray boxes indicate the terminal repeat/UTR regions.

To obtain our choice of *k*-mer length that was used as seeds for conserved sequence signature identification, covering the *Orthoflavivir*us genus, we used KITSUNE (28) to calculate ACF, representing the pairwise genome similarity based on *k*-mer profiles at a specific *k*-mer length. The individual *Flaviviridae* genomes were compared against the set of 10,472 reference genomes to determine whether the genomes had common *k*-mers or not. The number of genomes with k-mer sequences in common with the 105 genomes in *Flaviviridae* was recorded for each *k*-mer length between 9 and 51 and plotted for comparison (Fig. 2A). From the plot, we identified the SUK length of the *Flaviviridae* genomes, highlighting *Orthoflavivirus* queries. Each curve represents a single *Flaviviridae* genome; at each *k*-mer length, it reports the number of genomes in the full viral reference collection (including itself) that share ≥1 canonical *k*-mer. We found that at a *k*-mer length of 23, a specific k-mer can be found for the *Orthoflavivirus*. Considering the genomes of standard reference viruses (Fig. 2B), the *k*-mer length 31 onward yielded specific *k*-mers. From the plots (Fig. 2A and B), at the cut point of 51 orthoflaviviral genomes, we found that a *k*-mer length of 19 nt was the shortest k-mer length of the *Orthoflavivirus* genus that contained specific common *k*-mer(s) among them. Whereas starting from *k*-mer length of 21, the shared *k*-mer counts dropped below the panel size, indicating that longer *k*-mers were becoming too specific for capturing full-panel conserved-signature discovery. In total, 526,787 unique *k*-mers were identified for the 51 orthoflaviviral genomes. We found that the top two *k*-mers were commonly found in at maximum 20 genomes (Fig. 2C). To reduce the number of trivial sliding window duplicates, the unique *k*-mers were used as the seed for a compacted De Bruijn graph based on sequence overlapping among them to produce unitigs derived from the aggregation of *k*-mers within the close neighbors. We obtained 4,814 unitigs in total. Only 339 of these unitigs had sequences present in at least three orthoflaviviral genomes, and thus were designated high-confidence unitigs (Fig. 2D) and used for further steps. The high-confidence unitigs ranged from 19 to 41 bp with a maximum *k*-mer support of 23 and a maximum number of unitigs present in the genomes of 20.

### Conserved region(s) discovery and extraction

The high-confidence unitigs were then mapped back to the 51 orthoflaviviral genomes using exhaustive, exact alignment with Bowtie. The vast majority of unitigs mapped to one or more loci in the reference panel without mismatches, confirming that the compacted De Bruijn graph-derived sequences faithfully represented *k*-mers present in the underlying genomes. Conservation scores were calculated by aggregating 300 bp windows from orthoflaviviral genomes. The distribution of the scores over pseudo-genomic coordinates is illustrated in Fig. 2E. At the cut-off of 15 genomes, four 300 bp windows within the pseudo-genomic coordinates were identified, two of which were 0–0.3 and 10.5–10.8 kb untranslated region/terminal repeats based on the genomic annotation results, and were neglected. Therefore, the two windows of 8.7–9.0 and 9.0–9.3 kbp were combined as the conserved region for the probe/primer design. Notably, the 600 bp conserved region is located on non-structural protein 5 (NS5).

### Primers and probe design for TaqMan RT-qPCR

**Fig 3:**
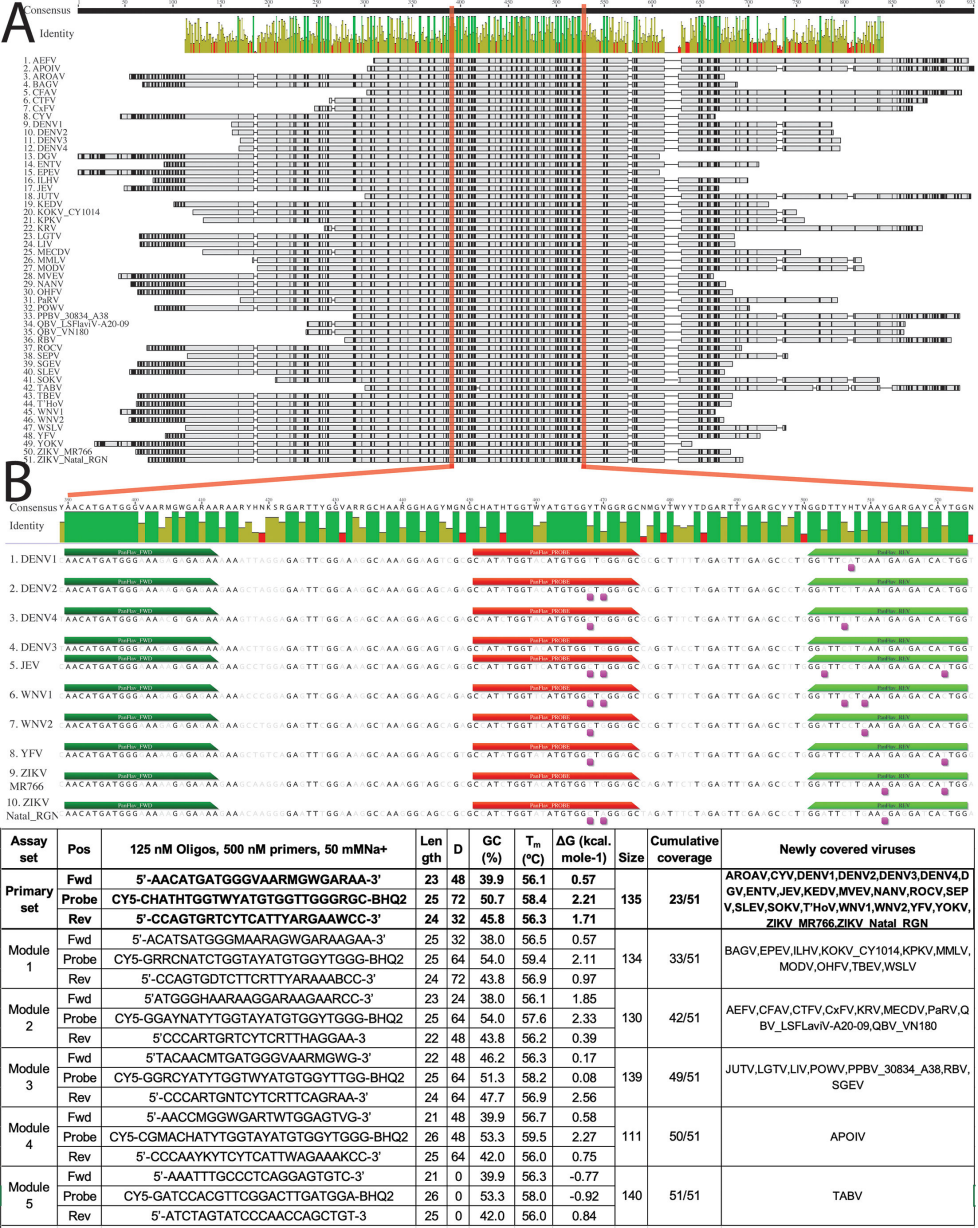
Alignment-free conserved-signature discovery and localization in *Orthoflavivirus* genomes. (**A**) MAFFT multiple sequence alignment of 600 bps conserved windows from 51 *Orthoflavivirus* genomes, the red lines indicate the start and the end of the amplicon window used to place the primers and probe. The amplicon region shows high per-base identity, indicated by multiple, contiguous, or near-contiguous conserved bases at 100% identity, colored in forest green. (**B**) The top panel shows MSA of the considered genomes, including dengue virus (DENV1–4), Japanese encephalitis virus (JEV), West Nile virus (WNV1, 2), yellow fever virus (YFV), and Zika virus (ZIKV) strains MR766 Natal RGN annotated with primer pair and probe position with mismatches in magenta underneath the genomes. The summary of the assay sets presented in the lower panel as a table shows chemical properties of each oligonucleotide, including probe and primer concentration, Na^+^ concentration, length, degeneracy, GC content, salt-corrected *T*_m_, Δ*G* of the reaction, product size, cumulative coverage, and newly covered viruses.

The sequences of the identified 600 bp conserved regions of the 51 individual orthoflaviviral genomes were extracted then multiple sequence alignments were performed using MAFFT software. The result is illustrated in Fig. 3A. The consensus sequence derived from the multiple sequence alignments has a length of around 930 bp, including some sequence blocks of conservation (forest green color on identity lane, indicating 100% identity). We next carefully inspected the multiple sequence alignment result using Geneious Prime software to find a region that can produce an amplicon size ~150 bp containing complement-specific target DNA sequences at both 3′ and 5′-end that are conserved across the species to initiate PCR amplification. We found a region in the middle, between positions 380 and 530 in the alignment, containing a dense block of conserved sequence that could be the target for an RT-qPCR primer-probe design (region between the two red vertical lines in Fig. 3A)

To reduce the complexity of the primer-probe design, we focused only on a subset of eight medically important orthoflaviviral arboviruses—DENV1–4, ZIKV, JEV, WNV, and YFV. The selected region contains sufficient conserved stretches to place primers and probes while accommodating the observed diversity (Fig. 3B). We designed a primer-probe set (Fig. 3B, lower panel) that possibly can be used to detect all eight of the considered viral species. The set was constrained to avoid mismatches or degeneracy in the last nucleotides of the forward primer and in the central portion of the probe, while permitting limited internal degeneracy or mismatches in the reverse primer when necessary to retain coverage. We observed some sequence variation across different genomes distributed on the complement sequences of the primers and probes; therefore, nucleotide degeneration of the primers and probes was iteratively applied based on the sequence variation context to result in a maximum mismatch of two (Fig. 3B, top panel) with an appropriate *T*_m_ and Δ*G*. The details of the designed primer-probe set are illustrated in Fig. 3B (lower panel). The primer-probe set used in the assay comprises a 23 nt forward primer, a 24 nt reverse primer, and a 25 nt hydrolysis probe (5′-Cy5, 3′-BHQ2), yielding a 135 bp amplicon. Across the three oligonucleotides, GC content ranged from 39.9% to 50.7%, and predicted *T*_m_ under the specified reaction conditions (125 nM oligos, 500 nM primers, and 50 mM Na^+^) were tightly matched (forward, 56.1°C; reverse, 56.3°C; and probe, 58.4°C; adjusted ranges shown in the figure). Thermodynamic estimates further indicated favorable hybridization (Δ*G* = 0.57–2.24), consistent with stable target recognition. We then checked whether the primer-probe set could be used to detect other orthoflaviviruses in the selected region *in silico* using Geneious Prime software. With two mismatches allowed, we found that the primer-probe set can possibly detect 15 Orthoflaviviruses (see Fig. S3A). Furthermore, we checked the specificity of the primers and probe sequences on the NCBI non-redundant nucleotide database by performing nucleotide blast (BLASTN) with two mismatches allowed and found 26,466 hits. Almost every hit was a known *Orthoflavivirus*. We deduplicated by TaxIDs, resulting in 42 records. Guapiaçu virus was represented by multiple accessions (TaxID = 2602438), and Panmunjeom flavivirus was represented by accession KY072986.1 (TaxID = 1928710) (see Table S3A and B). Interestingly, these two species were flaviviruses newly isolated from mosquitoes (41, 42) that have not been fully taxonomically classified; however, we hypothesize that they are in the *Orthoflavivirus* genus.

We also designed additional primer-probe sets for the 28 *Orthoflavivirus* viruses that were not predicted to be detected by the primary assay *in silico* on the same conserved region. Following the same criteria, five additional primer-probe sets were required in combination with the primary set to achieve predicted detection coverage of all 51 *Orthoflavivirus* viruses. These modular add-on sets are shown in Fig. 3B lower panel and Fig. S3B through F, with full oligonucleotide properties and species coverages provided in Table S4A through E.

### Performance evaluation of the designed primer-probe set for TaqMan RT-qPCR assay

**Fig 4:**
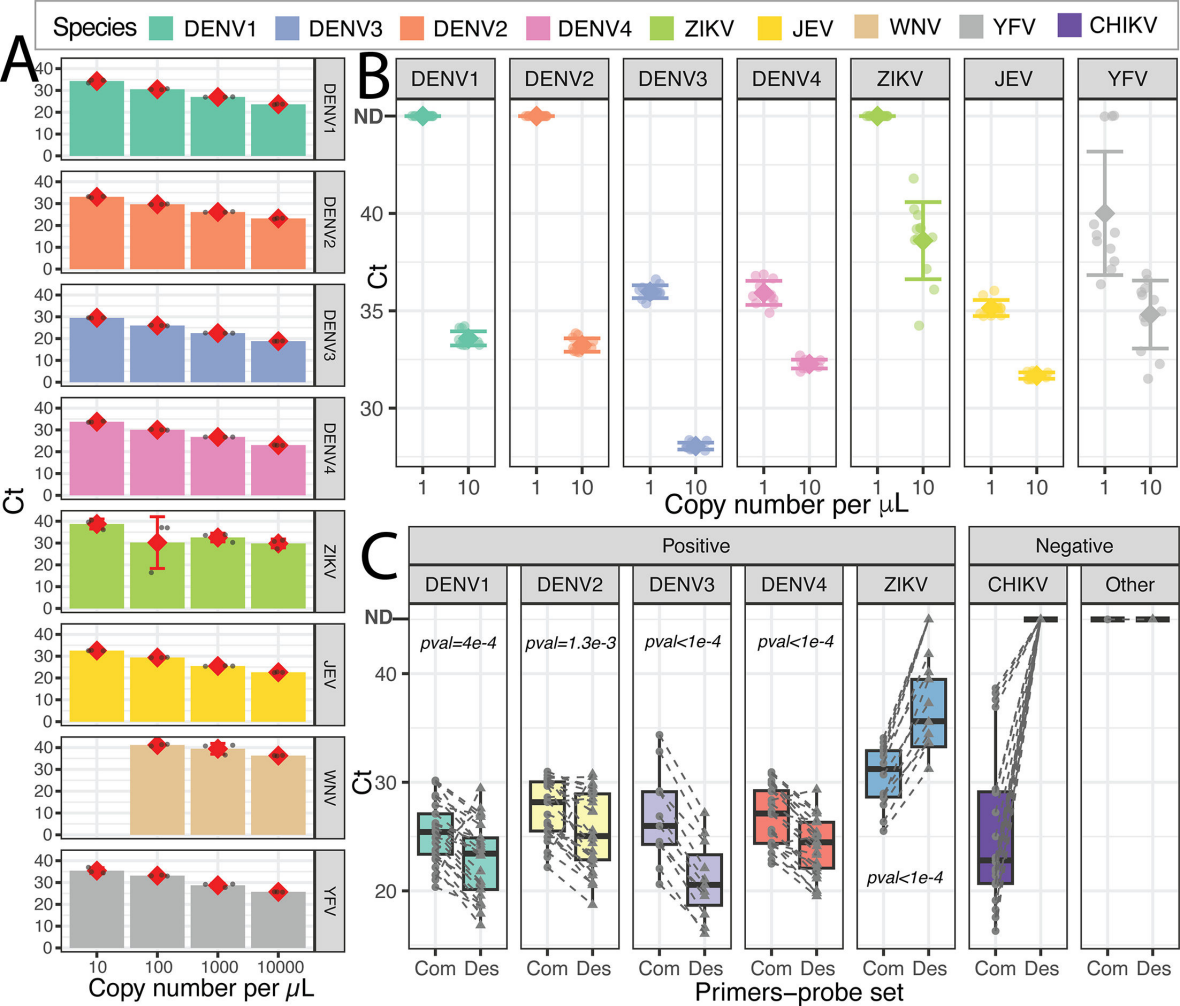
Alignment-free conserved-signature discovery and localization in *Orthoflavivirus* genomes. (**A**) Detection capability of the designed primer-probe set using reference nucleic acids of medically important arboviruses, including dengue virus serotypes 1–4 (DENV1–4), Zika virus (ZIKV), Japanese encephalitis virus (JEV), West Nile virus (WNV), and yellow fever virus (YFV). Ct values were evaluated across serial input concentrations ranging from 1 to 10,000 copies/µL, depending on sample availability. DENV1–4, ZIKV, JEV, and YFV were detected within a range of 1–10,000 copies/µL. Points represent individual reactions, red diamonds indicate mean Ct values, and error bars indicate ± SD. (**B**) Limit of detection (LOD) assessment of the designed primer-probe set. LOD testing for DENV1–4, ZIKV, and JEV was performed at 1, 10, 100, and 1,000 copies/µL using 12 replicates per dilution. For YFV, initial screening experiments were performed in triplicate at 100–10,000 copies/µL, followed by additional testing at 1 and 10 copies/µL for YFV using 12 replicates per dilution. The LOD was defined as the lowest concentration yielding positive amplification in >95% of replicates. Points represent individual reactions, red diamonds indicate mean Ct values, and error bars indicate ± SD. ND, not detected. (**C**) Paired comparison of Ct values between assays in clinical samples. Ct values obtained with the commercial kit (Com; circles) and the designed primer-probe set (Des; triangles) are shown for each target, with paired measurements from the same sample connected by dashed lines, for 100 positive samples and 50 positive samples (20 positive with Chikungunya virus [CHIKV] and 30 others). Box plots summarize the distributions (center line, median; box, interquartile range; and whiskers, range). Relative to the commercial kit, the designed primer-probe set detected DENV1–4 significantly earlier (lower Ct; *P* = 0.0004 [*n* = 25], 0.0013 [*n* = 25], <0.0001 [*n* = 11], and <0.0001 [*n* = 25] for DENV1–4, respectively) but detected ZIKV later (higher Ct; *p* < 0.0001 [*n* = 14]). *P* values were calculated using a two-sided paired *t*-test on matched Com–Des Ct values for each target. Overall diagnostic performance is reported in Tables 1 and 2.

First, we performed a pilot experiment to test the capability of the designed primer-probe set with the standard reference samples of the medically important arbovirus at an arbitrary number of copies/µL PCR reaction, depending on all the available materials. As illustrated in Fig. 4A, the designed primer-probe set was able to detect DENV1–4, ZIKV, JEV, and YFV within a range from 1 to 10,000 copies/µL in all replicates. For WNV, amplification was detected reliably at higher concentration, within a range of 1,000–10,000 copies/µL.

Before applying the designed primer-probe set on clinical samples, the LOD of the primer-probe was determined using the nucleic acids of seven arboviruses, excluding WNV due to inconsistent amplification in the pilot screening experiments. For DENV1–4, ZIKV, JEV, the LOD evaluation was performed at 1, 10, 100, and 1,000 copies/μL in 12 replicates per dilution. The LOD evaluation of YFV and WNV included initial screening experiments performed in triplicate (100–10,000 copies/μL), followed by an additional 12 replicates at 1 and 10 copies/μL when applicable. The LOD, defined as the lowest concentration yielding positive amplification in >95% of replicates. The assay LODs for DENV3, DENV4 were 1 copy/μL; for DENV1, DENV2, YFV, and ZIKV, 10 copies/μL (Fig. 4B; Table S5). The %CV of arbovirus detection was below 6% in all cases.

Repeatability and reproducibility experiments evaluated the precision of the assay. The nucleic acids of the seven arboviruses (DENV1–4, ZIKV, JEV, and YFV) at concentrations of 1, 10, 100, and 1,000 copies/μL were used to assess the intra-assay repeatability in 12 replicates, except for YFV at 100 and 1,000 copies/μL, for which 3 replicates were used. The intra-assay repeatability results showed CVs below 2% for the detection of DENV1–4 and JEV at all dilutions. Similar results were seen for ZIKV at 100 and 1,000 copies/μL, whereas CVs were approximately 5% at 1 and 10 copies/μL. For YFV, CVs ranged from approximately 3–5% at 1 and 10 copies/μL. Inter-assay reproducibility was evaluated between two operators across 5 days using a total of 20 replicates at high (1,000 copies/μL) and low (100 copies/μL) concentrations. The assay also showed the CVs less than 5% in all viruses at both dilutions, ranging from 1.15% to 3.20% for DENV4 and JEV detection at high concentration (1,000 copies/μL) (Table 1).

**TABLE 1:** Intra-assay repeatability and inter-assay reproducibility of the designed primer-probe set^*a*^

Pathogens	Intra-assay repeatability								Inter-assay reproducibility			
	1,000 Copies/μL		100 Copies/μL		10 Copies/μL		1 Copy/μL		1,000 Copies/μL		100 Copies/μL	
	Detected/ replicates)	Mean (SD, % CV)	Detected/ replicates	Mean (SD, % CV)	Detected/ replicates	Mean (SD, % CV)	Detected/ replicates	Mean (SD, % CV)	Detected/ replicates	Mean (SD, % CV)	Detected/ replicates	Mean (SD, % CV)
DENV1	12/12	26.35 (0.12, 0.46)	12/12	30.05 (0.19, 0.62)	12/12	33.58 (0.36, 1.09)	0/12	–	20/20	27.33 (0.73, 2.65)	20/20	31.09 (0.76, 2.45)
DENV2	12/12	26.20 (0.30, 1.14)	12/12	29.13 (0.15, 0.50)	12/12	33.24 (0.34, 1.04)	1/12	42.54 (ND, ND)	20/20	26.79 (0.54, 2.02)	20/20	30.43 (0.66, 2.16)
DENV3	12/12	20.62 (0.11, 0.55)	12/12	24.19 (0.30, 1.22)	12/12	28.05 (0.18, 0.63)	12/12	35.98 (0.33, 0.92)	20/20	20.71 (0.48, 2.33)	20/20	24.26 (0.41, 1.67)
DENV4	12/12	25.18 (0.17, 0.68)	12/12	28.49 (0.21, 0.74)	12/12	32.26 (0.23, 0.71)	12/12	35.92 (0.62 1.72)	20/20	25.65 (0.30, 1.15)	20/20	29.15 (0.41, 1.39)
ZIKV	12/12	32.52 (0.30, 0.93)	12/12	35.89 (0.29, 0.82)	12/12	38.60 (1.98, 5.13)	3/12	40.47 (1.89, 4.67)	20/20	33.79 (0.62, 1.84)	20/20	37.68 (0.63, 1.66)
JEV	12/12	25.29 (0.31, 1.24)	12/12	29.01 (0.17, 0.59)	12/12	31.67 (0.16, 0.52)	12/12	35.14 (0.41, 1.17)	20/20	26.05 (0.83, 3.20)	20/20	29.52 (0.89, 3.02)
YFV	3/3	28.72 (0.87, 3.04)	3/3	33.14 (0.23, 0.7)	12/12	34.81 (0.75, 5.02)	9/12	38.34 (1.16, 3.02)	NT	NT	NT	NT

^*a*^ %CV, percentage coefficient of variation; DENV, dengue virus; JEV, Japanese encephalitis virus; ND, not determined; NT, not tested; SD, standard deviation; YFV, yellow fever virus; ZIKV, Zika virus; –, no positive replicates were detected: mean Ct, SD, and %CV were not estimated.

In addition, analytical specificity was evaluated in terms of cross-reactivity using other pathogens, including influenza A virus strain A/PR/8/34 (ATCC; VR-95), RSV strain B (ATCC; VR-1580), CHIKV strain ROSS, enterovirus 71 (EV-71) strain H (ATCC; VR-1432), cytomegalovirus (CMV) strain AD-169 (ATCC: VR-538), hepatitis C virus (HCV), hepatitis B virus (HBV), human immunodeficiency virus (HIV) strain AE, *Orientia tsutsugamushi* strain KARP (accession number SAMN51290297), *Rickettsia typhi* strain SI-typh0423 (accession number SAMN51290298), *Plasmodium falciparum* strain 3D7 (ATCC; PRA-405D), *Leptospira interrogans* serovar Copenhageni strain Fiocruz L1-130 (ATCC; BAA-1198D-5), *Escherichia coli* strain Seattle 1946 (DSM 1103, NCIB 12210); ATCC: 25922, *Staphylococcus aureus* strain Seattle 1945 (ATCC: 25923), and *Pseudomonas aeruginosa* (ATCC: 27853). The assay showed no cross-reactivity with other pathogens (Table S6).

A total of 150 archived clinical plasma samples were used to assess the clinical performance of the assay. These included 100 orthoflavivirus-positive samples and 50 negative samples collected between 2018 and 2024. At the time of collection, samples were diagnosed using commercial kits. Pathogen-specific real-time RT-qPCR results generated using the designed primer-probe set were compared with those obtained from the commercial kits for each individual sample (Fig. 4C). The overall performance of the designed primer-probe was 97.33% accuracy (95% CI, 93.31–99.27%), 96% sensitivity (95% CI, 90.07–98.90%), and 100% specificity (95% CI, 92.89–100%) (Table 2). For DENV1–4, our designed primer-probe showed 100% sensitivity (95% CI, 71.51–100%) and specificity (95% CI, 97.09–100%), while for ZIKV it showed 71.43% sensitivity (95% CI, 41.28–100%) and 100% specificity (95% CI, 97.32–100%) (Table 2). Comparing the Ct value with the commercial kit, the overall Ct value from the designed primer-probe was significantly earlier to detect DENV1–4 (*P* = 0.0004, 0.0013, <0.0001, and <0.0001 for detection of the four DENV strains, respectively) but later to detect ZIKV (*P* ≤ 0.0001) (Fig. 4C). Bland-Altman analysis demonstrated strong concordance between two assays for DENV3 (mean ΔCt: −5.77, limit of agreement [LoA]: −8.12 to −3.42) (Fig. S4E), and ZIKV detection (mean ΔCt: 7.057, LoA: 3.10 to 11.01) (Fig. S4I). Most data points were distributed within the LoA, showing good consistency between the methods. However, only 4% (1 out of 25) of Ct values for DENV2, as well as 8% (2 out of 25) for DENV1 and DENV4, fell outside the 95% LoA (DENV1: −8.46 to 3.42 [Fig. S4A]; DENV2: −7.39 to 3.39 [Fig. S4C]; and DENV4: −5.99 to 0.396 [Fig. S4G]). The correlation for pan-flaviviruses amplification obtained by two assays was moderate for DENV1 (*r* = 0.5486; *P* = 0.0045) (Fig. S4B) and DENV2 (*r* = 0.6514; *P* = 0.0004) (Fig. S3D), while highly correlated for DENV3 (*r* = 0.9695; *P* ≤ 0.0001) (Fig. S4F), DENV4 (*r* = 0.8114; *P* ≤ 0.0001) (Fig. S4H), and ZIKV (*r* = 0.8081; *P* = 0.0047) (Fig. S4J).

**TABLE 2:** Clinical performance of the designed primers-probe

Pathogens	The number of samples			TP	FN	TN	FP	Sensitivity (%) (95% CI)	Specificity (%) (95% CI)	PPV (%) (95% CI)	NPV (%) (95% CI)	Accuracy (%) (95% CI)
	Positive	Negative	Total									
Pan-*Orthoflavivirus*	100	50	150	96	4	50	0	96.00 (90.07–98.90)	100 (92.89–100)	100 (96.23–100)	92.59 (82.71–97.03)	97.33 (93.31–99.27)
DENV1	25	125	150	25	0	125	0	100 (86.28–100)	100 (97.09–100)	100 (86.28–100)	100 (97.09–100)	100 (97.57–100)
DENV2	25	125	150	25	0	125	0	100 (86.28–100)	100 (97.09–100)	100 (86.28–100)	100 (97.09–100)	100 (97.57–100)
DENV3	11	139	150	11	0	139	0	100 (71.51–100)	100 (97.38–100)	100 (71.51–100)	100 (97.38–100)	100 (97.57–100)
DENV4	25	125	150	25	0	125	0	100 (86.28–100)	100 (97.09–100)	100 (86.28–100)	100 (97.09–100)	100 (97.57–100)
ZIKV	14	136	150	10	4	136	0	71.43 (41.90–91.61)	100 (97.32–100)	100 (69.15–100)	97.14 (93.69–98.73)	97.33 (93.31–99.27)

## DISCUSSION

The global spread of *Orthoflavivirus* pathogens, including DENV, ZIKV, JEV, WNV, and YFV, presents a complex diagnostic challenge, as overlapping symptoms make clinical differentiation impossible. While RT-qPCR remains the standard for acute-phase molecular diagnosis, species-specific assays can become resource-intensive when multiple viruses must be tested and may lose inclusivity as mutations accumulate at primer-probe binding sites. The traditional approach focuses on designing primer-probe sets specific to individual species. In contrast, a pan-*Orthoflavivirus* primer-probe set is intended as a broad initial screening tool for surveillance and early detection when *Orthoflavivirus* infection is suspected but the causative species is unknown. Fewer primer-probe sets may reduce assay cost and complexity compared with testing multiple species-specific RT-qPCR assays, while positive samples can be further characterized using species-specific RT-qPCR, sequencing, or other confirmatory methods for more accurate identification. This study proposes a systematic top-down AF computational framework that uses comparative genomic results using existing viral genomic data from public databases to produce a pan-viral genus detection assay. Our study adopted an AF *k*-mer strategy by decomposing genomes into fixed-length substrings (*k*-mers) and compared them with exact string statistics, unlike approximation results derived from biological sequence alignment. This approach treats the sequences as an unordered bag of features (*k*-mers), allowing for the rapid, unbiased processing of thousands of genomes to identify specific and conserved sequence “seeds” of the considered genomes for primer-probe design.

The need for modular add-on primer-probe sets was particularly evident for highly divergent viruses such as the Tamana bat virus (TABV). TABV was previously considered a tentative species proposed for assignment to a new genus (40) and has recently been suggested to form a separate clade within *Flaviviridae* (43). Although TABV was included in the original design panel based on the taxonomic annotations available at the time of data set construction, current taxonomy assigns it to the genus *Tamanavirus* rather than the core *Orthoflavivirus* clade (44). Consistent with this, TABV occupied a distant position relative to the main Orthoflavivirus cluster (Fig. 1D) and showed reduced compatibility with the primary conserved primer-probe region. Consequently, an additional TABV-compatible module was required to achieve complete predicted coverage of the viruses included in our analysis. This finding supports our modular design strategy, demonstrating that highly divergent members may fall outside the practical inclusivity range of a single broadly reactive primer-probe set. This also demonstrates the importance of taxonomic information and the robustness of the approach.

A primary limitation of this *k*-mer-guided approach, compared to traditional MSA-based analysis, is that it does not model positional homology across the entire genome. Instead, it is specifically designed to identify locally conserved, *k*-mer-supported regions with high potential for primer-probe placement. While this strategy reduces computational complexity and circumvents the challenges of aligning highly diverse viral genomes, it cannot replace MSA for evolutionary, phylogenetic, or recombination analyses. Furthermore, because *k*-mer conservation relies on exact or near-exact sequence matches, biologically homologous regions that exceed the divergence threshold at the selected *k*-mer length may be overlooked. Consequently, downstream local MSA inspection remains essential to evaluate sequence variation, guide degenerate primer-probe placement, and validate the suitability of candidate regions for RT-qPCR assay design.

Analysis of 51 orthoflaviviral genomes revealed three regions of high conservation (Fig. 2E), including 5′ and 3′ untranslated regions (UTRs), which are replete with complex RNA secondary structures—stem-loops and dumbbells—that are vital for viral replication, cyclization, and host factor binding (45–47). The 3′ UTRs are also the birthplace of subgenomic flaviviral RNA (sfRNA), a small, non-coding RNA produced when the host exoribonuclease (XRN1) degrades the viral genome in a 5′-to-3′ direction but stalls at the rigid pseudoknots of the 3′ UTR (48). This results in the accumulation of 3′ UTR fragments at a level that can exceed that of genomic RNA by orders of magnitude (49). While targeting the 3′ UTR can yield highly sensitive assays (due to the abundance of targets), it introduces biological noise. By choosing NS5 over the UTRs, our study opts for stoichiometric accuracy (measuring the actual virus genome) and broad inclusivity over the raw signal amplification offered by the sfRNA pool. This decision is central to understanding the sensitivity discrepancies observed in the clinical data, particularly for the Zika virus.

Our designed primer-probe set demonstrated high analytical sensitivity, detecting 1–10 copies/μL of standard viral reference material without cross-reactivity against non-target pathogens. The reproducibility was also high with %CV values less than 5.5% between assays, which is important when applying our assay in routine lab settings. However, the LOD experiments were performed using serially diluted extracted nucleic acid rather than intact virus spiked into a clinical matrix prior to extraction. Therefore, the reported LOD values should not be interpreted as true clinical sensitivity. Future validation using known quantities of virus spiked into negative clinical matrices, followed by extraction and RT-qPCR, will be necessary to account for extraction efficiency, matrix-associated inhibition, and potential RNA loss during sample processing.

Our designed primer-probe set demonstrated superior performance compared to the commercial kit for DENV serotypes, yielding significantly earlier Ct values. However, the lower sensitivity observed for ZIKV represents a limitation of the current assay. However, storage-related RNA degradation, which has been previously reported (50–52), may have contributed to the missed detection in archived ZIKV-positive samples. Other variables, including low initial viral RNA concentrations, primer-probe compatibility, amplification efficiency, and the selection of the NS5 gene over UTR-associated targets, likely contributed to this discrepancy. Further evaluation using freshly collected ZIKV-positive specimens is required to determine the relative impact of these factors. Nevertheless, the implementation of this primer-probe set can substantially reduce assay costs for broad pan-viral surveillance via affordable custom synthesis. Ultimately, large-scale validation incorporating additional viral strains and clinical specimens from diverse geographic regions is necessary to confirm the robust performance of this pan-*Orthoflavivirus* assay.

In summary, we demonstrated the systematic top-down approach by an AF method, *k*-mer-guided design for TaqMan RT-qPCR primer-probe design for pan-viral detection, enabling the ability to accurately detect multiple viruses in a considered *genus* rather than just the *species*. The workflow can be applied to other groups of viral pathogens of interest. In an interconnected world where the next pandemic threat is only a flight away, such broad-spectrum surveillance tools are the bedrock of global biosecurity.

## SUPPLEMENTAL MATERIAL

Supplemental figures jcm.00388-26-s0001.docx Figures S1 to S4.

Table S1A jcm.00388-26-s0002.xlsx The detail of the archived clinical samples (positive).

Table S1B jcm.00388-26-s0003.xlsx The detail of the archived clinical samples (negative).

Table S2A jcm.00388-26-s0004.xlsx All viral genomes used in the study.

Table S2B jcm.00388-26-s0005.xlsx The detail of orthovirus genomes used in the study.

Table S3A jcm.00388-26-s0006.xlsx Summary of the results of In silico validation of the design probe/primer based on NCBI non redundant database.

Table S3B jcm.00388-26-s0007.xlsx Representative of taxid from table s3A, ucovering newly 2 orthoviruses that have not classified in the database.

Supplemental tables jcm.00388-26-s0008.docx Tables S4 to S6.

ASM does not own the copyrights to Supplemental Material that may be linked to, or accessed through, an article. The authors have granted ASM a non-exclusive, world-wide license to publish the Supplemental Material files. Please contact the corresponding author directly for reuse.
