# Supplementary material for: Alignment-free k-mer-guided design of a pan-Orthoflavivirus RT-qPCR assay
Source: J Clin Microbiol. 2026 Jul 27;64(8):e00388-26. doi: 10.1128/jcm.00388-26 (PMC13463809; doi:10.1128/jcm.00388-26)
Supplement: Supplementary file 1 [file jcm.00388-26-s0001.docx]

**Supplementary Figures**

A

B

C

D

**Supplementary Fig. S1. Copy number quantification by standard curves**

**(A)** Ten-fold serial dilutions of the kit positive-control template were used to generate standards for RT-qPCR quantification. **(B-D)** Singleplex standard curves and representative amplification plots for dengue virus **(B)**, Zika virus **(C)**, and Japanese encephalitis virus (JEV) **(D)** values were regressed against log_10_ (copies per reaction) to derive standard curve equations for copy number estimation.

**Supplementary Fig. S2 Complete phylogenomic tree for family *Flaviviridae* with virus names of the genus *Orthoflavivirus* written on tips**

A

**Supplementary Fig. S3** (continued on the next page)

B

**Supplementary Fig. S3** (continued on the next page)

C

**Supplementary Fig. S3** (continued on the next page)

D

**Supplementary Fig. 3** (continued on the next page)

E

F

**Supplementary Fig. S3** (continued on the next page)

**Supplementary Fig. S3. In silico primer–probe evaluation across *Orthoflavivirus* for all candidate sets**

With up to two mismatches allowed, the primer–probe set (A) was predicted to detect 13 additional pathogenic genomes, including Bussuquara virus (AROAV), Chaoyang virus (CYV), Donggang virus (DGV), Entebbe bat virus (ENTV), Sepik virus (SEPV), ,Kedougou virus (KEDV), Murray Valley encephalitis virus (MVEV), Nanay virus (NANV), Sepik virus (SEPV), Saint Louis encephalitis virus (SLEV), Sokoluk virus (SOKV), T'Ho virus (T’HoV), and Yokose virus (YOKV).

Collectively, across candidate sets (A–F), the union of predicted detections covered all 51 Orthoflavivirus genomes in the reference panel under the same mismatch criterion

| A |  | B |  |
| --- | --- | --- | --- |
| C |  | D |  |
| E |  | F |  |
| G |  | H |  |
| I |  | J |  |

**Supplementary Fig. S4** Bland-Altman and Pearson’s correlation plots, Pearson correlation coefficient (r) and *p-value* (p), **(A)** Bland-Altman plots of dengue virus serotype 1 (DENV1); **(B)** Pearson’s correlation of DENV1; **(C)** Bland-Altman plots of DENV2; **(D)** Pearson’s correlation of DENV2; **(E)** Bland-Altman plots of DENV3; **(F)** Pearson’s correlation of DENV3; **(G)** Bland-Altman plots of DENV4; **(H)** Pearson’s correlation of DENV4; **(I)** Bland-Altman plots of Zika virus (ZIKV); **(J)** Pearson’s correlation of ZIKV. The dotted horizontal black line shows the mean of the difference (bias), and the dotted horizontal red line represents the lower and upper 95% LoA (bias ± 1.96 × SD).
