# Supplementary material for: Alignment-free k-mer-guided design of a pan-Orthoflavivirus RT-qPCR assay
Source: J Clin Microbiol. 2026 Jul 27;64(8):e00388-26. doi: 10.1128/jcm.00388-26 (PMC13463809; doi:10.1128/jcm.00388-26)
Supplement: Supplementary file 2 [file jcm.00388-26-s0008.docx]

**Supplementary Table 4**

(A-D) Chemical properties (oligos and salt concentration, length, degeneracy, GC content, T_m_ of salt-adjusted condition, ΔG, and amplicon size) of the 5 additional modular primer-probe sets for the 28 Orthoflaviviruses that cannot be detected by the primary assay.

**Supplementary Table 4A**

| Pos | 125 nM Oligos, 500 nM primers, 50 mMNa+ | Length | D | GC (%) | T_m_ (Adjusted) | ΔG (kcal. mole-1) | Product size | Detectable  viruses |
| --- | --- | --- | --- | --- | --- | --- | --- | --- |
| Fwd | 5’-ACATSATGGGMAARAGWGARAAGAA-3’ | 25 | 32 | 38 | 56.5ºC  (54.0 – 59) | 0.57 | 134 | AROAV,BAGV,CYV,DENV2,DENV3,DENV4,DGV,EPEV,ILHV,KEDV,KOKV_CY_1014,KPKV,MMLV,MODV,OHFV,QBV_VN180,ROCV,SEPV,SOKV,TBEV,T’HoV,WNV2,WSLV,YOKV,ZIKV_MR766 |
| Probe | CY5-GRRCNATCTGGTAYATGTGGYTGGG-BHQ2 | 25 | 64 | 54 | 59.4ºC  (54 – 64.7) | 2.11 |  |  |
| Rev | 5’-CCAGTGDTCTTCRTTYARAAABCC-3’ | 24 | 72 | 43.8 | 56.9ºC  (51.2 – 60.7) | 0.97 |  |  |

**Supplementary Table 4B**

| Pos | 125 nM Oligos, 500 nM primers, 50 mMNa+ | Length | D | GC (%) | T_m_ (Adjusted) | ΔG (kcal. mole-1) | Product size | Detectable  viruses |
| --- | --- | --- | --- | --- | --- | --- | --- | --- |
| Fwd | 5’ATGGGHAARAAGGARAAGAARCC-3’ | 23 | 24 | 38 | 56.1ºC  (52.6 – 60.2) | 1.85 | 130 | AEFV,CFAV,CTFV,CxFV,KRV,MECDV,MODV,PaRV,QBV_LSFlaviV-A20-09,QBV_VN180,RBV |
| Probe | CY5-GGAYNATYTGGTAYATGTGGYTGGG-BHQ2 | 25 | 64 | 54 | 57.6ºC  (53.0 – 62.3) | 2.33 |  |  |
| Rev | 5’CCCARTGRTCYTCRTTHAGGAA-3’ | 22 | 48 | 43.8 | 56.2ºC  (51.3 – 61.8) | 0.39 |  |  |

**Supplementary Table 4C**

| Pos | 125 nM Oligos, 500 nM primers, 50 mMNa+ | Length | D | GC (%) | T_m_ (Adjusted) | ΔG (kcal. mole-1) | Product size | Detectable  viruses |
| --- | --- | --- | --- | --- | --- | --- | --- | --- |
| Fwd | 5’TACAACMTGATGGGVAARMGWG-3’ | 22 | 48 | 46.2 | 56.3ºC  (51.6 – 60.9) | 0.17 | 139 | AROAV,BAGV,DGV,ILHV,JEV,JUTV,KEDV,KPKV,LGTV,LIV,MMLV,MODV,MVEV,OHFV,POWV,PPBV_30834_A38,RBV,ROCV,SEPV,SGEV,SLEV,SOKV,TBEV,WNV1,WNV2,WSLV,ZIKV_MR766,ZIKV_Natal_RGN |
| Probe | CY5-GGRCYATYTGGTWYATGTGGYTTGG-BHQ2 | 25 | 64 | 51.3 | 58.2ºC  (52.9 – 63.4) | 0.08 |  |  |
| Rev | 5’-CCCARTGNTCYTCRTTCAGRAA-3’ | 24 | 64 | 47.7 | 56.9ºC  (51.7 – 62) | 2.56 |  |  |

**Supplementary Table 4D**

| Pos | 125 nM Oligos, 500 nM primers, 50 mMNa+ | Length | D | GC (%) | T_m_ (Adjusted) | ΔG (kcal. mole-1) | Product size | Detectable viruses |
| --- | --- | --- | --- | --- | --- | --- | --- | --- |
| Fwd | 5’-AACCMGGWGARTWTGGAGTVG-3’ | 21 | 48 | 39.9 | 56.7ºC  (51.9 – 60.3) | 0.58 | 111 | APOIV,JUTV,SGEV |
| Probe | CY5-CGMACHATYTGGTAYATGTGGYTGGG-BHQ2 | 26 | 48 | 53.3 | 59.5ºC  (54.9 – 64.4) | 2.27 |  |  |
| Rev | 5’-CCCAAYKYTCYTCATTWAGAAAKCC-3’ | 25 | 64 | 42 | 56.0ºC  (51.7 – 60.7) | 0.75 |  |  |

**Supplementary Table 4E**

| Pos | 125 nM Oligos, 500 nM primers, 50 mMNa+ | Length | D | GC (%) | T_m_ (Adjusted) | ΔG (kcal. mole-1) | Product size | Detectable viruses |
| --- | --- | --- | --- | --- | --- | --- | --- | --- |
| Fwd | 5’-AAATTTGCCCTCAGGAGTGTC-3’ | 21 | 0 | 39.9 | 56.3ºC | -0.77 | 140 | TABV |
| Probe | CY5-GATCCACGTTCGGACTTGATGGA-BHQ2 | 26 | 0 | 53.3 | 58ºC | -0.92 |  |  |
| Rev | 5’-ATCTAGTATCCCAACCAGCTGT-3 | 25 | 0 | 42 | 56.ºC | 0.84 |  |  |

**Supplementary Table 5** The LOD of the designed primer-probe set on the 6 arboviruses

| Pathogens | (Detected/ replicates) | Mean  (SD, % CV) | LOD |
| --- | --- | --- | --- |
| DENV1 | 12/12 | 33.58 (0.36, 1.09) | 10 copies/µL |
| DENV2 | 12/12 | 33.24 (0.34, 1.04) | 10 copies/µL |
| DENV3 | 12/12 | 35.98 (0.33, 0.92) | 1 copiesµL |
| DENV4 | 12/12 | 35.92 (0.62 1.72) | 1 copies/µL |
| ZIKV | 12/12 | 38.60 (1.98, 5.13) | 10 copies/µL |
| JEV | 12/12 | 35.14 (0.41, 1.17) | 1 copies/µL |
| YFV | 12/12 | 34.81 (0.75, 5.02) | 10 copies/µL |
| WNV | 3/3* | 35.39 (2.49, 1.03) | 10,000 copies/µL |

*WNV LOD testing was performed using screening experiments in triplicate at 10000, 1000, and 100 copies/µL. Positive amplification was observed in 3/3 replicates at 10000 copies/µL, 2/3 replicates at 1000 copies/µL, and 0/3 replicates at 100 copies/µL.

**Supplementary Table 6** The cross-reactivity test of primer-probe

| Pathogen reference strains | Strains/ sources | Copy number  (Ct from commercial qPCR kit) | Detected/ replicate |
| --- | --- | --- | --- |
| Chikungunya virus (CHIKV) | strain ROSS | 6.79×10^4^ copies/µL  (Ct 21.318) | 0/1 |
| Influenza A virus | strain A/PR/8/34 (ATCC VR-95) | 4.88×10^4^ copies/µL  (Ct 26.55) | 0/1 |
| *Orientia tsutsugamushi* | strain KARP  Accession number SAMN51290297 | 1.57×10^4^ copies/µL  (Ct 26.152) | 0/1 |
| Enterovirus 71 (EV-71) | strain H (ATCC VR-1432) | 3.29×10^3^ copies/µL  (Ct 27.952) | 0/1 |
| *Escherichia coli* | FDA strain Seattle 1946 [DSM 1103, NCIB 12210]; ATCC: 25922 | 1.89×10^3^ copies/µL  (Ct 23.42) | 0/1 |
| *Staphylococcus aureus* | Seattle 1945; ATCC: 25923 | 1.64×10^3^ copies/µL  (Ct 22.80) | 0/1 |
| *Pseudomonas aeruginosa* | ATCC: 27853 | 9.49×10^2^ copies/µL  (Ct 24.29) | 0/1 |
| Respiratory syncytial virus (RSV) | strain B (ATCC VR-1580) | 8.13×10^2^ copies/µL  (Ct 29.93) | 0/1 |
| Cytomegalovirus (CMV) | strain AD-169 (ATCC: VR-538) | 3.99×10^2^ copies/µL  (Ct 26.46) | 0/1 |
| *Rickettsia typhi* | strain SI-typh0423  Accession number SAMN51290298 | 2.13×10^2^ copies/µL  (Ct 26.44) | 0/1 |
| *Plasmodium falciparum* | strain 3D7 (ATCC PRA-405D) | 2.05×10^2^ copies/µL  (Ct 27.91) | 0/1 |
| *Leptospira interrogans*  serovar Copenhageni | strain Fiocruz L1-130 (ATCC BAA-1198D-5) | 1.55×10^2^ copies/µL  (Ct 27.69) | 0/1 |
| Human Immunodeficiency Virus (HIV) | strain AE | Approx 1.39×10^5^ copies/mL* | 0/1 |
| Human Immunodeficiency Virus (HIV) |  | Approx 1.39×10^4^ copies/mL* | 0/1 |
| Hepatitis C virus (HCV) VL1488 |  | viral load 49,300 IU/mL | 0/1 |
| Hepatitis C virus (HCV) VL1537 |  | viral load 18,000 IU/mL | 0/1 |
| Hepatitis B virus (HBV) VL0086 |  | viral load 2,600 IU/mL | 0/1 |
| Hepatitis B virus (HBV) VL0057 |  | viral load 2,550 IU/mL | 0/1 |

* HIV was 3-4 log diluted from 1.39×10⁸ copies/mL (Ct 14.88).
